# Phenology and Cover of Plant Growth Forms Predict Herbivore Habitat Selection in a High Latitude Ecosystem

**DOI:** 10.1371/journal.pone.0100780

**Published:** 2014-06-27

**Authors:** Marianne Iversen, Per Fauchald, Knut Langeland, Rolf A. Ims, Nigel G. Yoccoz, Kari Anne Bråthen

**Affiliations:** 1 Department of Arctic and Marine Biology, UiT The Arctic University of Norway, Tromsø, Norway; 2 Norwegian Institute for Nature Research (NINA), Department of Arctic Ecology, Fram Centre, Tromsø, Norway; Dauphin Island Sea Lab, United States of America

## Abstract

The spatial and temporal distribution of forage quality is among the most central factors affecting herbivore habitat selection. Yet, for high latitude areas, forage quantity has been found to be more important than quality. Studies on large ungulate foraging patterns are faced with methodological challenges in both assessing animal movements at the scale of forage distribution, and in assessing forage quality with relevant metrics. Here we use first-passage time analyses to assess how reindeer movements relate to forage quality and quantity measured as the phenology and cover of growth forms along reindeer tracks. The study was conducted in a high latitude ecosystem dominated by low-palatable growth forms. We found that the scale of reindeer movement was season dependent, with more extensive area use as the summer season advanced. Small-scale movement in the early season was related to selection for younger stages of phenology and for higher abundances of generally phenologically advanced palatable growth forms (grasses and deciduous shrubs). Also there was a clear selection for later phenological stages of the most dominant, yet generally phenologically slow and low-palatable growth form (evergreen shrubs). As the summer season advanced only quantity was important, with selection for higher quantities of one palatable growth form and avoidance of a low palatable growth form. We conclude that both forage quality and quantity are significant predictors to habitat selection by a large herbivore at high latitude. The early season selectivity reflected that among dominating low palatability growth forms there were palatable phenological stages and palatable growth forms available, causing herbivores to be selective in their habitat use. The diminishing selectivity and the increasing scale of movement as the season developed suggest a response by reindeer to homogenized forage availability of low quality.

## Introduction

The spatial and temporal distribution of forage quality is regarded as one of the most central factors affecting herbivore habitat use [1], [2]. Forage quality is distributed spatially in terms of composition of species of differing palatability [3]–[5], and temporally in terms of plant phenology with plants being more palatable when younger [6]–[9]. However, variability in plant phenology caused by local environmental factors and species-specific physiology and life history, are also major contributors to spatial patterns in forage quality [10]–[13]. Species-specific phenological development is often related to plant traits, such as capacity for nutrient acquisition, storage and tissue resistance [10]. Plants with similar morphological and/or physiological traits defining plant growth forms [14], show similar phenological strategies [11], [15]. The importance of growth forms as a source of spatial variation in phenology is particularly pronounced in arctic and alpine ecosystems, which are characterized by a broad spectrum of plant growth forms that shift their dominance relations according to environmental factors [16]. Plant phenology has proved to be important for herbivore migration and offspring production [17]–[22]. Here we ask to what extent plant phenology at the functional level of growth forms, is important for predicting large herbivore habitat selection across spatial and temporal scales.

A common feature of food resources is that quality and quantity are often inversely correlated [23], [24], with the most nutritious tending to be the least common [25]. This is the case for many high latitude ecosystems where less palatable heaths dominate and nutrient-rich forage is more scattered [26], [27]. A much-discussed trade-off faced by large ruminants is thus the selection of high quality forage versus forage abundance. Although plant quality is an essential factor in forage selection among herbivores, high latitude studies have found quantity to be more important than quality [28], [29]. This can be explained by a generally high plant quality at high latitudes because of cold climatic conditions, or at least seasonally so [30]. Another factor influencing the relationship between quality and quantity might be that less palatable species often have slower phenological development compared to the more palatable species [10], [11], [15]. This might imply a seasonal change in foraging patterns with use of the generally less palatable species, but still young and more abundant species early in the season.

For studying forage quality at an extensive scale, grouping plant species into a limited number of functional groups has been advocated [14], [31], [32]. In cold biomes a functional grouping based on growth forms is often used in studies on plant communities' responses to environmental conditions [14], [26], [33]. Plant growth forms differ in their nutrient value and palatability for herbivores [4], [7], [21], [34], [35] and they show consistent differences in phenology at alpine– and high latitude areas [11]. Hence, growth forms may be useful for studying forage quality at an extensive spatial and temporal scale. Using plant growth forms as predictors of herbivore habitat use also provide a way to link plant herbivore interactions to ecosystem functions and services [36]. For instance, growth form traits are related to growth rate, amount of resistant tissues and nutrient content [14], which are core properties for ecosystem productivity, transpiration and nutrient cycling [14], [37] and essential for ecosystems responses to climate change [16]. Hence, assessing the importance of growth forms for extensive herbivore habitat use represents one way of accomplishing the dual goal of understanding habitat use and ecosystem effects.

Extensive scale studies are considered as crucial for understanding both herbivore foraging decisions [38] and their ecosystem effects [39]. Telemetry has made large-scale studies on herbivore habitat use feasible [40], [41], but telemetry studies are still challenging as collection of data on resource availability is hard to accomplish unless surrogates for resource availability such as NDVI are employed [42]. Moreover, it is challenging to choose the proper scale(s) of herbivore habitat use [41], for instance between the scale of the forage bite and the scale of the forage distributional range. Hence, whereas extensive studies on herbivore habitat use are advancing due to technological developments such as telemetry, methodological challenges still pose limitations on to what questions can be answered on habitat use.

The reindeer (*Rangifer tarandus tarandus L.*) is an abundant, highly mobile large herbivore in tundra ecosystems that constantly move while foraging during the summer [43]. In the present study we investigate to what extent phenology and cover of growth forms are predictive to the habitat selectivity of reindeer throughout a summer season. The study extends a spatial scale of 400 km^2^ in a low-arctic reindeer pasture district where low-palatable dwarf shrubs are dominating. To meet the methodological challenges, we first sampled the phenology and cover of growth forms in *in situ* plots randomly distributed along the movement tracks of reindeer, as close to the real-time passing of the animals as possible. Secondly, we used First-Passage Time (FPT) analyses [44] to investigate the scales of reindeer movement and how the scales changed as the summer season advanced. Finally, to address the selection of forage quality and quantity, we investigated the relationship between the movement pattern measured as FPT and the forage cover and phenology in each plot. Specifically we asked if both plant phenology of less abundant palatable growth forms and non-palatable but dominant growth forms are predictors of reindeer habitat selection. We asked if plant phenology diminishes as a predictor for reindeer habitat selection as summer develops. Finally we asked if the scale of habitat selection by reindeer is similar throughout the season, indicative of high selectivity for plant phenology early in the season and continued selectivity for palatable growth forms as the summer season develops.

The aim of this study was threefold, reflecting not only the main biological question asked but also addressing methodological challenges of studies in habitat selection: 1) Investigate to what extent forage quality and quantity assessed at the level of growth forms predicts the habitat selection of a large ungulate at high latitude during summer, 2) elucidate the applicability of growth forms as an ecosystem relevant proxy for forage quality, and 3) develop the methodology pertinent to studies of habitat selection in large herbivores by combining the FPT methodology with *in situ* measurements of forage quality and quantity. We found habitat selectivity by reindeer to vary throughout the summer, a variability for which both phenology and cover of growth forms were significant predictors.

## Materials and Methods

### Ethics statement

Authorization from the ethics committee is for this study not applicable; Reindeer to which we attached GPS collars are not endangered or protected and are owned by Sami people that gave approval to the experiment. GPS collars are widely in use in reindeer husbandry in order to facilitate the overview of the whereabouts of the reindeer. Moreover, specific permission was not required for doing plant analysis at the locations given by the reindeer GPS positions: firstly the analysis was non-destructive and secondly these rangelands in Norway are common ground.

### Study area

**Figure 1:**
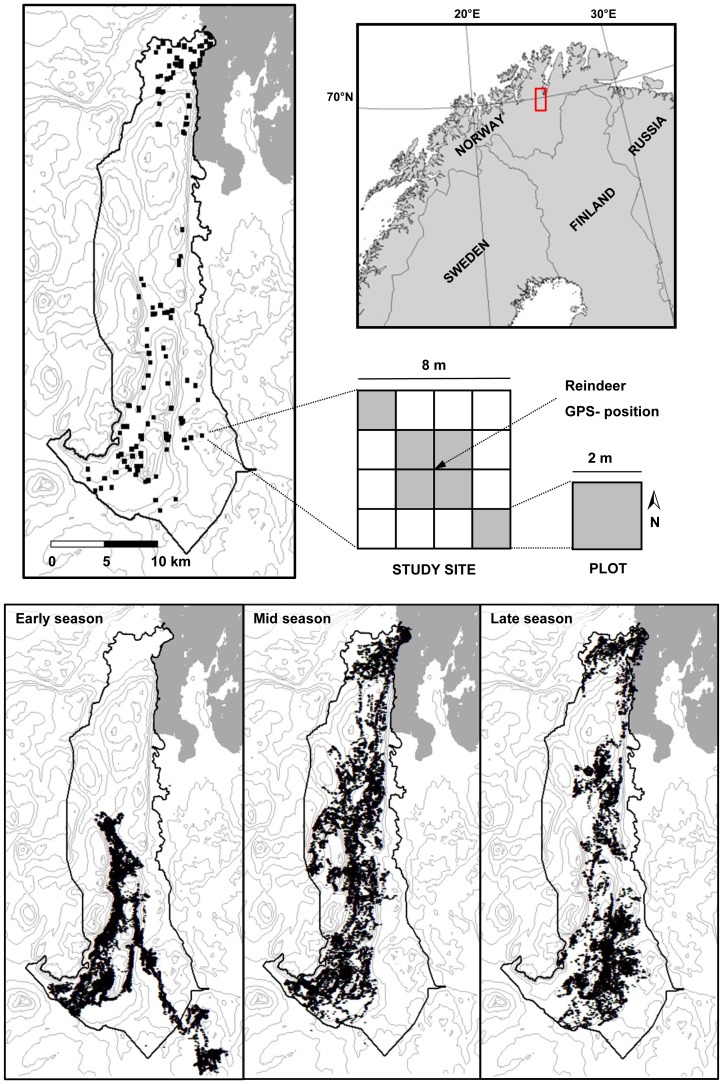
Study location. Upper panel: The location of the reindeer district in which the study took place along with a presentation of the hierarchical structure of the study design. A total of 144 sites (black squares) were located within the district by GPS-positions from analyses of tracking data from reindeer (see main text for more detail). Within five days of its localisation each site was analysed for the cover and plant phenology of growth forms. Analysis were conducted within six plots (shaded) of the site. Lower panel: The three maps show the positions of freely ranging reindeer in the early, mid and late season of the summer of 2006.

The study area is a reindeer management district located in Porsanger, Northern Norway, at 70°N, 22–23°E, covering an area of about 400 km^2^ (Figure 1), and constitutes a summer pasture for a herd of semi-domesticated reindeer with an average density across the district of 6–7 animals per km^2^ (estimated for 1980–2003, http://www.reindrift.no). The district belongs to the oceanic to continental section of the north-boreal and alpine zone [45]. Mean temperatures in July are in the range of 8–12°C and the length of the growing season varies between 110 and 130 days [45]. Permafrost is rare below 350–450 m asl. [46] and an active layer develops very soon after snow melt. The mean yearly precipitation lies between 400–700 mm. The snow disappears in the study area between May 14 and June 11 (for 2004–2007) (Norwegian Water Resources and Energy Directorate, http://snokart.nve.no).

The reindeer district stretches from the shores of the Porsangerfjord to high alpine areas at 1100 m asl., and is composed of a mixture of bedrock and sediment deposits of low to moderate to high rates of nutrient availability (Geological survey of Norway, http://www.ngu.no/en-gb/hm/Norwegian-geology/). Typical vegetation of the district is dwarf shrub and low shrub heaths dominated by *Empetrum nigrum, Vaccinium myrtillus*, and *Betula nana* (nomenclature follows The Panarctic Flora, http://nhm2.uio.no/paf/).

Common herbivores other than reindeer are ptarmigan (*Lagopus lagopus* and *L. muta*), hare (*Lepus timidus*) and small rodents such as Norwegian lemmings (*Lemmus lemmus*) and grey sided voles (*Myodes rufocanus*), domestic sheep (*Ovis aries*) and moose (*Alces alces*). In 2006 a considerable attack of geometrid moths (*Epirrita autumnata* and *Operopthera brumata*) in a mountain birch (*Betula pubescens*) forest affected a small part of the district.

The herding district consists of 5 herds that are separated during winter but share the summer pasture. The study herd is calving in a fenced area on the border of the district in May and let into the summer pasture to mix with the other herds after marking in early June. The other herds are calving inside the summer pasture area (see [47], [48] for a detailed description of the calving area and herding system in the district). Fences and natural barriers limit reindeer movement outside the district. The animals are actively herded only once during the summer when the calves from the other herds are marked for ownership. In 2006, when this study was carried out, this event took place during the last days of June and the first week of July hence this period is not included in our study. Apart from this event the animals were moving freely during the study period from June 6 to August 23 2006.

### Study design

Randomly selected female reindeer (n = 20) with calves were captured and marked with GPS collars (Televilt Tellus II GPS collars with VHF remote download of data using a Televilt RX-900 receiver unit, Followit Lindesberg AB, http://wildlife.followit.se/) on June 4, 2006 in collaboration with the reindeer owners. Until the collars were removed in mid-September, GPS positions were taken at 5-minute intervals. Nine collars failed during the season, leaving track data from 11 animals available for this study.

Plant analyses were conducted daily in three periods during which the reindeer were tracked; June14–26, July 8–28 and August 12–23. To select positions for plant analyses, GPS positions from the reindeer were downloaded daily using a VHF receiver. Because the positions were sampled with a constant time interval (5 min), most positions were sampled in preferred high-use areas; i.e. in areas where the animals had a low speed. Therefore, in order to ensure both high and low -use areas, we sampled a random position based on the animal's speed along the track: Each day the last 24 hours of track data were downloaded from the collars we could reach with the VHF receiver. For each of the downloaded animal-tracks the speed was calculated between successive positions. A uniform distribution defined by the track's minimum and maximum speed was calculated and one speed was randomly chosen. Finally, the position along the track where the animal had a speed closest to the randomly selected speed was selected as a site for plant analyses.

We aimed for daily downloading of GPS positions from as many reindeer as possible and as dispersed as possible. A total of 144 sites (see Table 1 for distribution per period) were visited for plant analyses from 1 to 4 days after the downloading of the tracks. Thus there was a lag of 2 to 5 days between when an animal was at a location and when a site was visited for collection of plant data.

**Table 1:** Overview of study sites and reindeer in the early, mid and late summer season.

Period	Sites (n)	Reindeer (n)	Estimates of habitat selection (n)	Estimates of habitat selection per site (mean [range])
**Early season**	26	11	87	3.35 [1]–[10]
**Mid season**	67	8	224	3.34 [1]–[7]
**Late season**	51	6	73	1.43 [1]–[3]
**Total season**	144	11	384	2.67 [1]–[10]

Number of sites for estimates of phenology and cover of plant growth forms, the associated number of reindeer from which we attained tracking data and the number of attained estimates of habitat selection (number of 7-day periods of reindeer tracks overlapping with sites in time and space), in total and per site. All data are presented for early, mid or late season and for the overall summer season.

### Collection of plant data

The sites for plant measurements consisted each of a 8×8 m^2^ square which were divided into 16 plots of 2×2 m^2^, with a selected GPS position (see above) defining its centre and with the direction of the plot towards the north (Figure 1). Six predefined plots were analysed in all sites for cover and vegetative phenology of six growth forms. The growth forms were evergreen dwarf shrubs, deciduous shrubs and deciduous dwarf shrubs, sedges (including rushes), grasses and herbs.

Percentage cover of growth forms was estimated visually with 10% increments in each plot. When just one or a few individuals of a growth form were present the growth form was given the cover value of 0.1%.

In each plot the phenology was measured on vegetative plant parts of one species representing a growth form. To randomise the selection of the plant to use for the phenology measure, a line was put down every 0.5 m inside the plot and for every growth form the first plant that hit the line was measured. The list of species encountered and measured is included in Table S1.

Determination of phenological stages are based on previous studies [6], [49], [50] but adjusted to fit all the species of the current study. Vegetative phenology for the deciduous shrubs was measured by scoring all the individual leaves of a selected branch to one of seven categories (see Table 2). The same scale was used for the evergreen shrubs but then on the new shoot of the year rather than single leaves (see [49]). In cases of variation of phenological stages between leaves on a shrub branch, the median stage was used. For graminoids the vegetative phenology was measured as the length of the longest, fresh leaf divided by the length of the longest withered leaf within the same ramet. The longest withered leaf was assumed to be the length of a phenological mature leaf. For forbs with the last year leaves missing, the longest fresh leaf was divided by the average maximum leaf length of withered leaves of all sampled individuals of the species.

**Table 2:** Definition of vegetative phenology for all growth forms.

Vegetative phenology: Shrubs and dwarf shrubs	
1	Leaf bud
2	Bursting bud
3	Recently burst leaf, light green
4	Completely burst leaf, young and light green
5	Darker green
6	Fully developed; dark green, thick leaves
7	Coloured leaves

Vegetative phenology: Sedges, grasses and herbs:	
	Length of longest living leaf/Length of longest dead leaf

Vegetative phenology was measured in two ways; in phenological stages for shrubs and dwarf shrubs, and as a continuous measure for graminoids and forbs. For shrubs and dwarf shrubs a branch defined by the lowermost branching point was used for measurements. For forbs and graminoids, an individual ramet was used for measurements.

Several calibration sessions were arranged during the field season to reduce observer bias in the estimations of cover and phenology [51]. The attack of the geometrid moths affected 7 out of the 144 sites. However, mainly one plant species was affected, the evergreen dwarf shrub *Empetrum nigrum*, and the affected sites were therefore retained for the data analysis.

### Data analysis

All statistical analyses were performed in the R environment (version 3.0.2, R Foundation for Statistical Computing, http://www.r-project.org). First-passage time analyses were programmed in Simula [52].

### Reindeer track data

**Figure 2:**
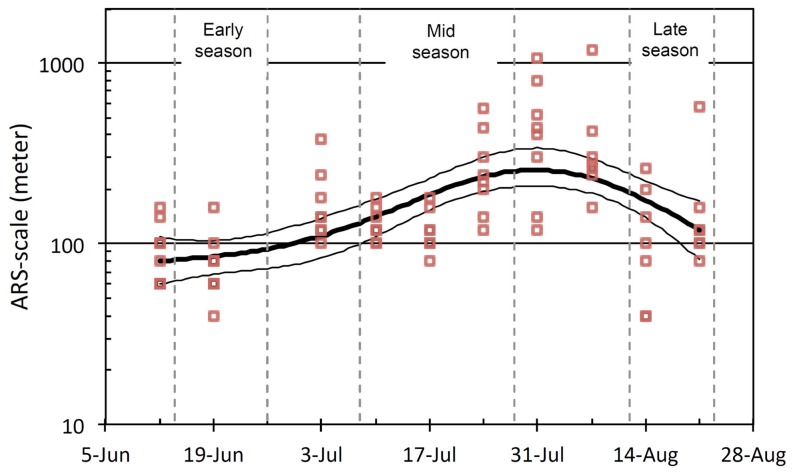
Change in the ARS-scale through the season. Grey bars are ARS-scales (i.e. the scale of the local maximum in variance of log FPT) for each 7-days period of the movement path of individual reindeer. The thick black line is the predicted ARS-scale from a gamm model using period as a predictor and log ARS-scale as a response. The thin black lines represent standard error.

We used First Passage Time (FPT) analyses of the GPS data to study the habitat use of reindeer. FPT is defined as the time required for an animal to cross a circle with a given radius and is a scale-dependent measure of how much time an animal spends within a given area [44]. As movement pattern was expected to change during the season, the path of each reindeer individual was segmented into 7-days periods and analysed with respect to FPT separately. To ensure that positions along the paths were equally represented [53], we interpolated positions to obtain a uniform distance interval of 20 m (see [54], [55]). Based on the interpolated positions, the variance in log-transformed FPT was calculated for radii ranging from 20 m to 1200 m with a 20 m increment. The radius giving the maximum variance in log FPT, has been termed the Area Restricted Search (ARS) -scale [53] (cf. Figure 2). It corresponds to the spatial scale at which the animal concentrates its time and is the scale that best differentiates between high and low passage time along the animal's path [54]. Accordingly, FPT-values were calculated at the observed ARS-scale for each reindeer in each period (cf. Figure 2), and were assigned to sites if their distance was less than the ARS-scale from a given site and their period overlapped a time lag of maximum 5 days preceding the plant analyses of the site. All FPT-values assigned to sites were averaged per reindeer per period to give a final sample unit of 384 for the habitat use analyses (Table 1).

We did not control for periods of resting or rumination. However, visual inspection of the data indicated that regular periods of inactivity were found scattered throughout the tracks, suggesting that the animals did not use specific places for rumination or resting. As long as resting and rumination takes place at regular intervals along the foraging track, these activities would not bias FPT as a measure of habitat use for foraging.

FPT-values increased throughout the summer, hence in order to compare habitat use responses between periods we standardized the FPT-values from each period by log10 transforming each value and then standardizing to mean zero and standard deviation equal to one.

### Plant data

**Figure 3:**
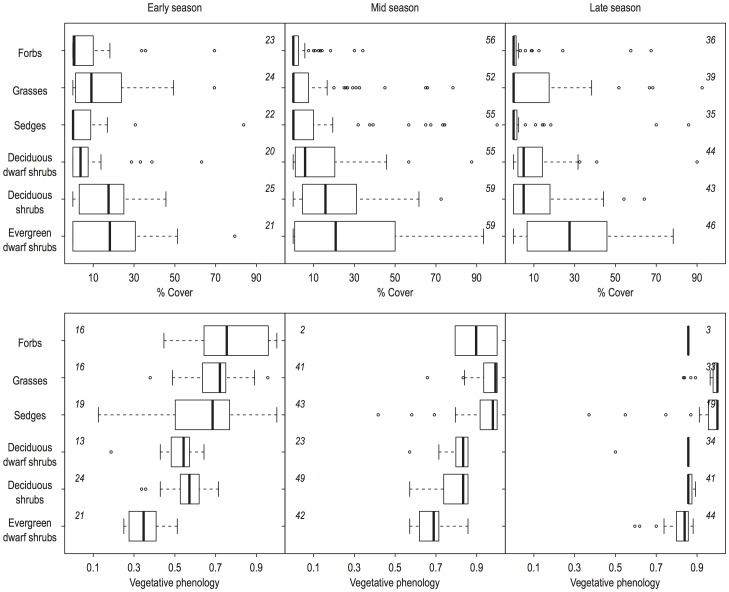
Cover and phenology of growthforms. Boxplots of a) cover (%) and b) vegetative phenology (presented on a relative scale, see Table 2 for actual scale) of each growth form in early, mid and late season. Numbers within panels represent number of sites for which a) the growth form was present and cover was estimated, or b) where phenological measurements of the growth form was possible.

Cover and phenology data for each growth form were averaged across all plots for each site before they were used as predictor variables. Phenology measurement scales for each growth form were transformed to a relative scale between 0 and 1 for making a comparison of the phenology between growth forms feasible (Figure 3).

### Modeling

First the ARS scale of the 7-days periods was analysed for its relation to time throughout the summer season with a mixed general additive model using the mgcv and nlme libraries in R [56]. Log ARS –scale was modelled with a smooth function with time as a fixed effect predictor and individual reindeer as a random component.

Then habitat selection with regard to forage quality and quantity was analysed with linear mixed effects models [57] using standardized FPT-values as response variable and vegetative phenology and cover of growth forms as fixed effects predictors. Individual reindeer was included as a random component. Separate models were run for the early, mid and late season periods.

**Figure 4:**
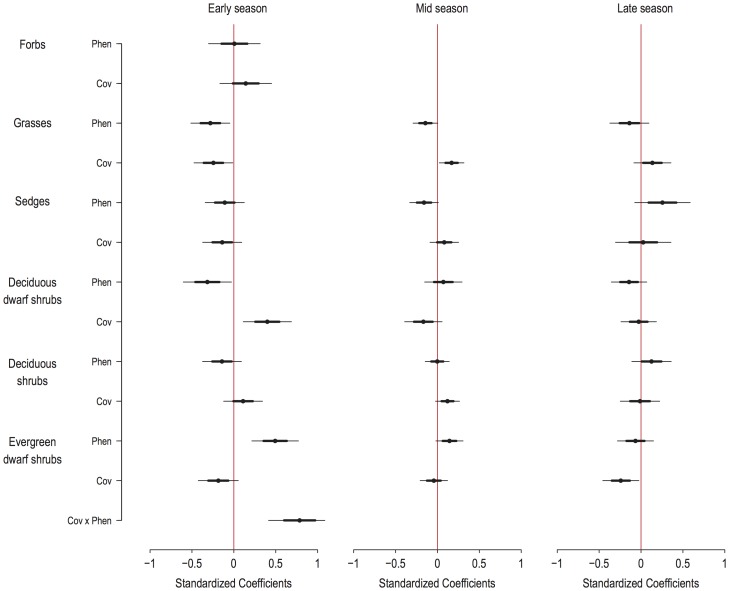
Standardized coefficients of the effects of phenology and cover of growth forms on FPT by reindeer. Relationships between phenology and cover of growth forms and FPT by reindeer are presented as standardized coefficients (see main text for explanation). Middle point give model estimate, thick lines give ±1 SE (approximately ±68% CI) and thin lines give ±2 SE (approximately ±95% CI). Predictor variables with thin lines that do not cross the central (red) line have a significant relationship to habitat selection by reindeer. Both negative and positive standardized coefficients are related to more selective habitat use by reindeer, with lower or higher values of a predictor variable respectively. For instance, for grasses, younger phenology give higher FPT values, indicating habitat selection for sites where grasses have younger phenology.

First we tested the predictive power of only cover in a model including all growth forms as predictors. Then we tested, for each growth form at a time, the multiplicative term between vegetative phenology and cover as a specific form for interaction. Because both variables were continuous, they were standardized (mean = 0, variance = 1) before modelling [58]. Whenever a product was non-significant it was left out of the model. Importantly, the output from the model testing the effect of cover of all growth forms, was marginally different from that of the models testing the effect of phenology and cover for each growth form separately (Figure 4, Table S2). We interpret this coherence between models as an indication of no confounding between the different growth forms in predicting habitat use. For visual presentation of results, the coefficients (i.e. standardized coefficients based on the scaled variables) were extracted from model outputs of the two-way interaction models.

## Results

### Seasonal patterns of reindeer area use

**Figure 5:**
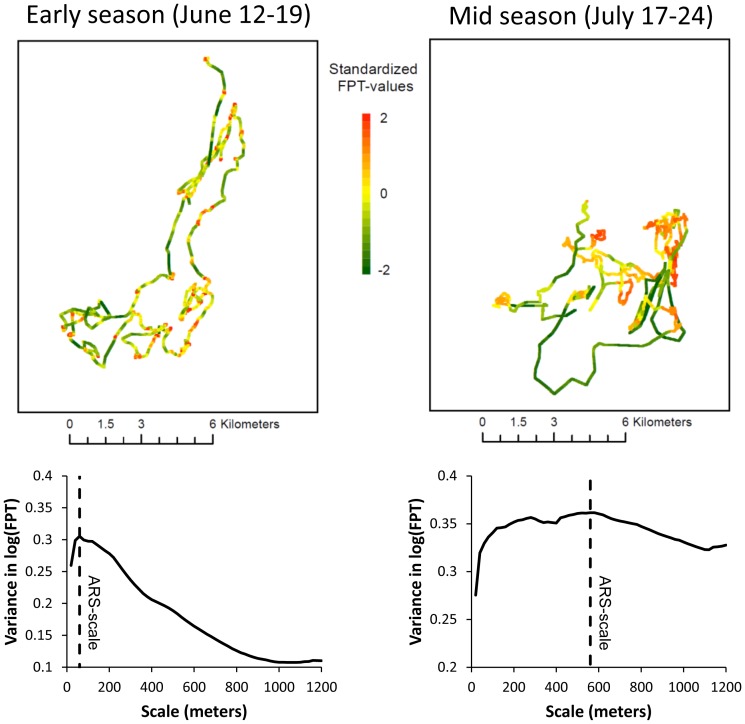
FPT-analyses. Upper panel: Two 7-days periods (second period of the early season and last period of the late season) of the path of the same individual (reindeer #35). Standardized FPT-values were calculated from the respective ARS-scales and are shown as green to red color along the tracks. Lower panel: Variogram of log-transformed FPT values for the two 7-days periods. The ARS-scale was defined at the local maximum in variance.

Reindeer were found to be present in most of the 400 km^2^ district in which the study was conducted (Figure 1). Still, the FPT analysis revealed that the way reindeer moved differed both temporally over the summer season and spatially across the district. The ARS-scales of the 7-days periods over the summer showed a non-linear change through the season (edf = 2.81, P<0.01, R^2^ (adj)  = 0.35; Figure 2, with individual reindeer SD = 0.15 and residual SD = 0.56). The ARS-scale increased from the beginning of June until the end of July and decreased by the end of the season. This suggests that the scale of the “intensive use” area (cf. Figure 5) increased as the summer developed (i.e. from about 80 m in the early season to 250 m in the mid season; Figure 2) and decreased to about 120 m in the late season.

### Seasonal patterns in growth form phenology and cover

The most available growth form to the reindeer along their tracks was the low palatable evergreen dwarf shrubs, followed closely by deciduous shrubs in the early and mid summer season, as judged from the average cover values of growth forms across sites (Figure 3). Grasses were the third most available growth form in the early summer season, after which it dropped in availability, whereas deciduous dwarf shrubs showed low but similar availability throughout the summer. The palatable forbs and the sedges were the least available growth forms (Figure 3).

The available phenology of growth forms for reindeer differed among the growth forms, especially in the early summer season when every growth form also had a range of phenological stages available over the sites (Figure 3). Evergreen dwarf shrubs were the least developed in the early season, with the average stage being light green, recently burst buds (Figure 3, Table 2). The deciduous shrubs and dwarf shrubs were more developed, with the average phenological stage being young and light green completely developed leaves. Finally, the phenology of sedges, grasses and forbs were the most developed, with the average available phenology having developed almost three quarters into phenological maturity (Figure 3). Whereas grasses and sedges reached full maturity by the mid summer season, shrubs and dwarf shrubs did not reach last stages until the late summer season (Figure 3). For the mid and late summer season hardly any forb species were accessible for phenological measures.

### Reindeer habitat selection predicted by growth form phenology and cover

Both the phenology and the cover for several of the growth forms were significant predictors of habitat selection (standardized FPT values) by reindeer, with predictions ranging from 25–75% of the total standard deviation of the estimated FPT. However, predictors differed in strength and changed between the seasons with the phenology of growth forms as a significant predictor in the early summer season only, whereas cover was significant throughout the summer (Figure 4, Table S2).

In the early season higher values of FPT (i.e. more use of an area) were associated to sites with younger phenology of grasses and deciduous dwarf shrubs, and older phenology of the evergreen dwarf shrubs (Figure 4), and to sites with more cover of deciduous dwarf shrubs. Moreover, higher values of FPT were associated with sites with both high cover and more advanced phenological stages of evergreen dwarf shrubs, as indicated by a significant product between phenology and cover for this growth form (Figure 4). For the mid season higher values of FPT were only significantly related to sites of higher grass cover, whereas for the late season only sites with lower cover of evergreen dwarf shrubs were selected for (Figure 4).

## Discussion

We found both forage quality and quantity assessed at the level of growth forms to predict habitat selection of a large ungulate at high latitude during summer. As anticipated, plant phenology of both less abundant palatable growth forms and non-palatable but dominant growth forms predicted habitat selection. The predictive effect of phenology was strong only in the early summer season, whereas habitat selection in response to cover was evident throughout the summer. However, the scale of habitat use by reindeer was not constant from the early to the late summer season. The ARS scale increased from about 80 to 250 m from early June to late July after which it again decreased until the end of the study. Also, whereas palatable growth forms were significant predictors in the early and mid-summer season, this was not the case in the late summer season when only selection for habitats with less cover of an unpalatable growth form was evident.

Availability of growth forms in habitats used by reindeer complied with the notion that quality and quantity of food resources often are inversely correlated [23], [24], with the most nutritious being the least common [25]. Less palatable growth forms were most abundant, with an average cover of 15–30% throughout the summer as opposed to an average cover of 0–5%, with one incidence of 10%, for the more palatable growth forms. Even the ranking of availability between growth forms was inversely correlated to palatability (evergreen dwarf shrubs > deciduous shrubs> deciduous dwarf shrubs > sedges > grasses > forbs, [43], [59]), and was the same as that estimated from a former study in the same district [26], where the choice of sites was completely random. Accordingly, because we in the present study estimated the availability from sites selected from the reindeer tracks, this indicates the availability of growth forms to reindeer to a large extent reflected the general composition of growth forms in the district. With low palatable growth forms dominating, we expected habitat usage to be selective because the most palatable growth forms would have to be used disproportionately to their availability (*sensu* [60]).

However, we found yet another inverse relationship between quality and quantity that could affect selectivity. That is, abundances of growth forms were inversely related to phenological development, with the most abundant growth forms being unpalatable and at young phenological stages and the least abundant growth forms being palatable and at older phenological stages. Because older phenological stages have less N and lower digestibility [6], [8], [17], whereas young phenological stages are the most nutritious, this relationship likely reduces difference in palatability between growth forms. Hence, whereas the relationship between availability and general palatability is likely to enforce selective habitat use for palatable growth forms, the relationship between availability and phenology is likely to cause selectivity also for less palatable species, diminishing differences in selectivity between growth forms. Accordingly in this study, we found selection for younger phenological stages of the more palatable growth forms and for later phenological stages of the least palatable but dominating growth form.

Whereas selectivity for young phenology early in the summer is in accordance with other studies on ungulates, (e.g. [17]) we are not aware of any studies showing selectivity for older phenology. As expected, a higher availability of the least palatable growth form strengthened the response to phenology as indicated by a positive effect of the product between cover and phenology. Such a switch in the direction of selectivity for phenological stages may have a simple explanation. That is, the average phenological stages of grasses and deciduous dwarf shrubs were past 50% maturity with selectivity for younger and assumable more nutritious stages. In contrast, selectivity for the evergreen dwarf shrubs was for the stage of recently attained full sized leaves. The more fully developed size of leaves is likely a prerequisite for the ability of reindeer to even access the new growth, considering that the evergreen dwarf shrubs were dominated by *Empetrum nigrum*, a species that has rather small leaves (on average 4.61 mm^2^ [61]). Moreover, the palatability of the fresh leaves of *E. nigrum* is probably related to the accumulation of phytotoxic substances that do not reach their maximum concentration until late in the summer season [62]. In line with this, there are anecdotal data indicating that reindeer rasp new shoots of *E. nigrum* in spring and early summer [63]–[65]. In general we found phenology of growth forms to be important as a predictor for habitat use only in the early summer season when the relationship between availability and phenology of growth forms was most evident.

We applied the FPT-method to reindeer movement patterns at a regional scale to estimate habitat use without *prior* assumptions on what spatial scale was relevant to reindeer [41]. Moreover, we did not need to choose any particular spatial scale, indicative of either distribution, home range, patch choice or food item (*sensu* [60]), or region, landscape and patch (*sensu* [38]), as all of these were integrated in the estimated area restricted search (ARS) and the associated FPT. Therefore, when areal extent of habitat use increased from early to mid season, our estimates of the predictive role of plant quality and quantity were sensitive to any differences in habitat selectivity also at the larger scale. The only spatial scale we chose was that of the sites for measures of forage availability. Ideally also the spatial scale of these plant data sampling sites should have matched the ARS scale at all times, but ARS scale calculations at the time of plant data collection was not possible for the current study.

We believe the choice of a method sensitive to the actual scale used by reindeer was instrumental to that we found both forage quality and quantity to be predictive of habitat selection. Even in the late summer season, when the reindeer used larger areas, we found less cover of evergreen dwarf shrubs to predict habitat selection. Also, the increased extent of land use by reindeer as the summer developed coincided with lower variability of phenology, both among and within growth forms, along with that the average cover of all growth forms but for the evergreen dwarf shrubs was less than 5%. Generally low availability of palatable growth forms corresponds to what has formerly been evaluated as a homogenization of forage resources found in districts of higher reindeer densities [26], and may restrain possibilities for selectivity causing animals to roam larger areas for food. There are however also other sources of explanation to the changes in habitat selection. For instance, seasonal changes in habitat use by caribou and reindeer has been linked to predation risk [66], insect harassment [67] and human disturbances [68] that are potentially also relevant here. Moreover, the decreasing extent of area use in the late season (Figure 2) might have coincided with mushroom availability, for which reindeer have a strong preference [69]. Nevertheless, in this study detailed estimates of forage quality and quantity rendered significant predictions for habitat selection as estimated from movements at a regional scale. Hence, we show that forage details measured at a scale similar to the patch scale (a scale for which forage quality is believed to be the sole predictor [38]), have relevance for habitat selection also at much larger scales.

Previously a preference for quantity to quality by reindeer at high latitudes has been documented [28], [29], whereas we found preference for both quantity and quality. We believe our contrasting result is related to our methodology. Whereas Van der Wal *et al*. [29] selected certain focal plant species as indicators of reindeer forage, we and Mårell *et al*. [28] included all species present and categorized them according to growth forms. By this approach we were able to include species that on their own were not dominating or common, but together with other species of the same growth form became abundant enough to be included in the statistical analysis. For instance the growth form of grasses constituted a total of 12 different species. We believe including all species made a difference to the ability to resolve the predictive role of forage to habitat use. And, although Mårell *et al*. [28] used growth forms, and a more sensitive measure to abundance (i.e. biomass as opposed to cover in our study), they applied broader growth form categories than in our study, possibly masking their ability to find growth form effects. Yet, and perhaps as important, we found clear indications of area restricted search (ARS) in the FPT-analysis of the movement patterns by reindeer, whereas this was not the case for Mårell *et al*. [28] although they also used a method sensitive to the actual movement patterns of reindeer (correlated random walk). With our extensive data set on movement patterns from GPS positions of 11 reindeer at 5 minutes intervals throughout the whole summer season, we achieved rigorous FPT-estimates of movement patterns of a wide-roaming animal like reindeer. Moreover, our approach analyzing sites of reindeer occurrence within 5 days of their visit was still enough to find a predictive role of both forage quality and quantity, probably because reindeer/caribou do not empty their forage resources at their feeding sites [70]. Hence, we believe our methodological approaches using FPT and plant growth forms were important to our success in finding a predictive role of both forage quality and quantity to reindeer habitat selection.

## Conclusions

Both forage quality and quantity were found as significant predictors to selective habitat use by reindeer, indicating selectivity for quality is also important in high latitude ecosystems where general forage quality is low. Early in the season palatable phenological stages and palatable growth forms were available, allowing reindeer to be selective in their habitat use. The diminishing selectivity later in the season reflected a homogenization of forage availability as the season developed, as there is probably a threshold in availability of palatable forage below which reindeer are no longer selective.

We see our methods applied as promising for future studies of habitat use and forage selectivity in mobile herbivores. We advocate the FPT-method as it admits the spatial scale of the study to be representative of the actual scale of the herbivore, and we advocate the use of plant growth forms as predictors of habitat use as they provide means for connecting herbivores to the ecosystem functions provided by their food resources.

## Supporting Information

Table S1 Overview of plant species and growth forms.
(DOCX)

Table S2 Effects of cover of growth forms on FPT-values of reindeer.
(DOCX)
