# Supplementary material for: Phenology and Cover of Plant Growth Forms Predict Herbivore Habitat Selection in a High Latitude Ecosystem
Source: PLoS One. 2014 Jun 27;9(6):e100780. doi: 10.1371/journal.pone.0100780 (PMC4074057; doi:10.1371/journal.pone.0100780)
Supplement: Table S1 — Overview of plant species and growth forms. (DOCX) [file pone.0100780.s001.docx]

### Supporting Information

**Table S1.** **Overview of plant species and growth forms encountered in this study**. Nomenclature follows The Panarctic Flora (<http://nhm2.uio.no/paf/>).

| **Latin name** | **Growth form** |
| --- | --- |
| *Agrostis capillaris* | Grass |
| *Agrostis mertensii* | Grass |
| *Agrostis stolonifera* | Grass |
| *Andromeda polifolia* | Evergreen dwarf shrub |
| *Anthoxanthum nipponicum* | Grass |
| *Avenella flexuosa* | Grass |
| *Arctostaphylos alpinus* | Deciduous dwarf shrub |
| *Betula nana* | Deciduous shrub |
| *Bistorta vivipara* | Forb |
| *Calamagrostis lapponica* | Grass |
| *Calamagrostis phragmitoides* | Grass |
| *Carex spp* | Sedge |
| *Carex aquatilis ssp aquatilis* | Sedge |
| *Carex bigelowii* | Sedge |
| *Carex nigra ssp nigra* | Sedge |
| *Carex rostrata* | Sedge |
| *Carex vaginata* | Sedge |
| *Chamaepericlymenum suecicum* | Forb |
| *Deschampsia cespitosa* | Grass |
| *Dryas octopetala* | Forb |
| *Empetrum hermaphroditum* | Evergreen dwarf shrub |
| *Eriophorum angustifolium* | Sedge |
| *Eriophorum vaginatum* | Sedge |
| *Festuca ovina* | Grass |
| *Festuca rubra* | Grass |
| *Festuca vivipara* | Grass |
| *Geranium sylvaticum* | Forb |
| *Juncus trifidus* | Sedge |
| *Ledum palustre* | Evergreen dwarf shrub |
| *Loiseleuria procumbens* | Evergreen dwarf shrub |
| *Luzula spicata* | Sedge |
| *Matteuccia struthiopteris* | Forb |
| *Nardus stricta* | Grass |
| *Pedicularis lapponica* | Forb |
| *Phyllodoce caerulea* | Evergreen dwarf shrub |
| *Salix glauca* | Deciduous shrub |
| *Salix herbacea* | Deciduous shrub |
| *Salix lapponicum* | Deciduous shrub |
| *Salix nigra* | Deciduous shrub |
| *Salix phylicifolia* | Deciduous shrub |
| *Solidago virgaurea* | Forb |
| *Trollius europaeus* | Forb |
| *Vaccinium vitis-idaea* | Evergreen dwarf shrub |
| *Vaccinium myrtillus* | Deciduous dwarf shrub |
| *Vaccinium uliginosum* | Deciduous dwarf shrub |
