# Supplementary material for: Phenology and Cover of Plant Growth Forms Predict Herbivore Habitat Selection in a High Latitude Ecosystem
Source: PLoS One. 2014 Jun 27;9(6):e100780. doi: 10.1371/journal.pone.0100780 (PMC4074057; doi:10.1371/journal.pone.0100780)
Supplement: Table S2 — Effects of cover of growth forms on FPT-values of reindeer. (DOCX) [file pone.0100780.s002.docx]

### Supporting Information

**Table S2. Effects of cover of growth forms on FPT-values of reindeer.**

Results (standardized coefficients ±2SE) from a linear mixed effect model on the relationship between the standardized FPT-values as response variable and the cover of the six growth forms as predictor variables for the early, mid and late summer season. The ID of reindeer collars was used as a random variable. See Figure 2 in main text for sample size of the different growth forms. Standardized coefficients in bold indicate significant relationship between FPT-values and cover.

|  | **Early season** | **Mid season** | **Late season** |
| --- | --- | --- | --- |
|  |  |  |  |
| **Forbs** | 0.010 ± 0.012 | 0.002 ± 0.019 | -0.003 ± 0.012 |
| **Grasses** | -0.006 ± 0.012 | **0.007 ± 0.006** | -0.003 ± 0.010 |
| **Sedges** | -0.006 ± 0.014 | 0.001 ± 0.005 | -0.002 ± 0.012 |
| **Deciduous dwarf shrubs** | 0.012 ± 0.014 | <0.001 ± 0.008 | -0.003 ± 0.012 |
| **Deciduous shrubs** | -0.010 ± 0.024 | 0.002 ± 0.006 | <-0.001 ± 0.013 |
| **Evergreen dwarf shrubs** | 0.008 ± 0.018 | <-0.001 ± 0.005 | **-0.011 ± 0.009** |
